# The single-cell atlas of the epididymis in mice reveals the changes in epididymis function before and after sexual maturity

**DOI:** 10.3389/fcell.2024.1440914

**Published:** 2024-08-05

**Authors:** Jiaxin Zhang, Ye Xie, Xiaoyan Wang, Yafei Kang, Chuxiong Wang, Qinying Xie, Xinyi Dong, Yonghong Tian, Donghui Huang

**Affiliations:** ^1^ Institute of Reproduction Health, Tongji Medical College, Huazhong University of Science and Technology, Wuhan, China; ^2^ Reproductive Center, Qingdao Women and Children’s Hospital, Qingdao Women and Children’s Hospital Affiliated to Qingdao University, Qingdao, China; ^3^ Department of Reproductive Endocrinology, Women’s Hospital, School of Medicine, Zhejiang University, Hangzhou, Zhejiang, China; ^4^ Shenzhen Huazhong University of Science and Technology Research Institute, Shenzhen, China

**Keywords:** single-cell RNA sequencing (sc-RNA seq), epididymis, clear/narrow cells, basal cells, principal cells

## Abstract

**Introduction:** The epididymis is important for sperm transport, maturation, and storage.

**Methods:** The head and tail of the epididymis of 5-week-old and 10-week-old C57 BL/6J male mice were used for single-cell sequencing.

**Results:** 10 cell types including main, basal, and narrow/clear cells are identified. Next, we performed cell subgroup analysis, functional enrichment analysis, and differentiation potential prediction on principal cells, clear cells, and basal cells. Our study indicates that the principal cells are significantly involved in sperm maturation, as well as in antiviral and anti-tumor immune responses. Clear cells are likely to play a crucial role in safeguarding sperm and maintaining epididymal pH levels. Basal cells are implicated in the regulation of inflammatory and stress responses. The composition and functions of the various cell types within the epididymis undergo significant changes before and after sexual maturity. Furthermore, pseudo-temporal analysis elucidates the protective and supportive roles of epididymal cells in sperm maturation during sexual maturation.

**Discussion:** This study offers a theoretical framework and forecasts for the investigation of epididymal sperm maturation and epididymal immunity.

## 1 Introduction

The single-cell RNA-sequencing method (scRNA-seq) is a novel technology utilized for the amplification and sequencing of the entire transcriptome at the individual cell level. The fundamental principle involves amplifying isolated single cells containing trace RNA and subsequently acquiring the expression profile of these cells through high-throughput sequencing. This method is predominantly employed for cell type identification and analysis of the spatio-temporal progression of cell development, thereby presenting new research opportunities in the field (Tanay and Regev, 2017). ScRNA-seq has found extensive application in reproductive research, encompassing but not limited to investigations in the uterus, testis, embryos, and other organs (Feng et al., 2022; Hong et al., 2022; Li et al., 2022a; Li et al., 2022b; Pique-Regi et al., 2022; Qian et al., 2022; Sacks et al., 2018; Wang et al., 2022; Zhou et al., 2022).

The epididymis is a vital male reproductive organ that plays a crucial role in the processes of sperm transport, maturation, and storage (Breton et al., 2019). Structurally, the epididymis is segmented into three distinct regions: the head, body, and tail (Johnston et al., 2005). Histologically, the epididymal lumen is lined with pseudostratified epithelium consisting of various cell types with diverse physiological functions that collectively regulate the microenvironment of the lumen. These cell types include main cells, clear/narrow cells, and basal cells (Sullivan et al., 2019), with some studies also identifying apical cells and halo cells (a subtype of T cells) (Martinez-Garcia et al., 1995). Various epididymal epithelial cells exhibit distinct gene expression profiles linked to specific physiological functions in various segments. The head and body primarily oversee sperm motility and fertilization capacity (Turner, 1995), whereas the tail predominantly facilitates sperm storage and protection (Jones and Murdoch, 1996). This segment-specific gene expression pattern forms a cohesive epididymal functional network crucial for sperm maturation (Johnston et al., 2005; Cornwall, 2009; Dacheux and Dacheux, 2014; Sullivan et al., 2019).

In a recent study, Rinaldi et al. (2020) utilized scRNA-seq to investigate epididymal epithelial cells in male FVB/NJ mice aged 10–12 weeks. They generated a comprehensive single-cell map, identifying various cell types such as master cells, clear cells, basal cells, and supporting cells, and characterized the segmental specificity of master cell populations. Additionally, the study revealed evidence of distinct subsets of stromal cell function within the epididymis and vas deferens. Sullivan et al. (2019) provided a detailed account of the efferent duct cells in the head of the human epididymis, highlighting their significance. Leir et al. (2020) emphasized the crucial function of CFTR in the normal transport of epididymal fluid. Additionally, Shi et al. (2021) conducted a study on cells from different regions of the mouse epididymis at two distinct time points, representing the initial wave and subsequent generation of sperm. Furthermore, Shi et al. (2021) not only observed temporal and spatial variations in cell composition and differentially expressed genes (DEGs) in the mouse epididymis but also identified two predominant cell subsets that may correspond to stereociliated cells. They also confirmed the elevated presence of mitochondria and mitochondrial transcriptome (MT) in both the epididymis and epididymis tail (Shi et al., 2021). Additionally, a separate study conducted in the laboratory delineated the cell map of the epididymis in individuals with 46, XY disorders of sexual development, revealing a significant increase in fibroblasts alongside notable decreases in main cells and basal cells (Shi et al., 2023).

Shi JW studied gene expression in 42-day and 56-day mouse epididymis, while Rinaldi, VD et al. mapped single cells in 10–12-week-old mice (Rinaldi et al., 2020; Shi et al., 2021). However, differences in epididymal epithelial cells before and after sexual maturity have not been detailed in any study. Subsequently, RNA-seq was conducted on murine epididymal epithelial cells to elucidate the gene expression profiles, functions, and variances pre- and post-sexual maturation across distinct epithelial cell subtypes. This investigation establishes a foundational framework and anticipates future research on epididymal sperm maturation and immunity.

## 2 Method

### 2.1 Animals

Male C57BL/6J mice were purchased from the animal center of Tongji Medical College (Wuhan, Hubei), housed under a 12 h dark, 12 h light cycle and with free access to food and water, treated in accordance with the guidelines of the Animal Research Committee and approved by the Centre of Experimental Animals of Huazhong University of Science and Technology (no. 42009800001411, 10-10-2015).

### 2.2 Tissue separation and preparation

Fresh tissue samples were preserved in sCelLiveTM tissue preservation solution (Singleron) on ice within 30 min of surgical extraction. The samples underwent three washes with Hanks balanced salt solution (HBSS), were subsequently sectioned into small pieces, and were digested with 3 mL of sCelLiveTM tissue separation solution (Singleron) from Singleron PythoN at 37°C for 15 min. Following digestion, cell suspensions were obtained and filtered through a 40 μm sterile filter. Subsequently, GEXSCOPE ^®^ RBC lysis buffer (RCLB, Singleron) was introduced, and the combination of cells and RCLB at a ratio of 1:2 (volume ratio) was allowed to incubate at room temperature for 5–8 min to eliminate red blood cells. Following this, the mixture underwent centrifugation at 300 g at 4°C for 5 min to separate the supernatant, which was then gently resuspended in PBS/DMEM. Ultimately, the specimen was treated with trypan blue and the viability of the cells was assessed using a microscope.

### 2.3 Sample collection and preparation

In this research, a total of five 5-week-old (5 W) and five 10-week-old (10 W) C57BL/6J mice were utilized. The mice were euthanized via cervical dislocation while under anesthesia, followed by the removal of epididymal fat and rapid separation of the epididymis under an anatomical microscope. Each sample was divided into two parts: head and tail. The freshly dissected tissue was preserved in sCelLiveTM tissue preservation solution (Singleron) on ice. The specimens underwent three washes with Hanks balanced salt solution (HBSS), were subsequently sectioned into small fragments, and then subjected to digestion with Singleron PythoN’s 3 mL sCelLiveTM tissue separation solution (Singleron) at 37°C for a duration of 15 min. Following this process, cell suspensions were obtained and passed through a 40 μm sterile filter. Subsequently, GEXSCOPE ^®^ RBC lysis buffer (RCLB, Singleron) was introduced, and the combination of cells and RCLB at a ratio of 1:2 (volume ratio) was allowed to incubate at room temperature for 5-8 min to eliminate red blood cells. Following this, the mixture underwent centrifugation at 300 g at 4°C for 5 min to separate the supernatant, which was then gently resuspended in PBS/DMEM. Ultimately, the specimen was treated with trypan blue and the viability of the cells was assessed using a microscope. Finally, five samples in each group were mixed into one sample.

### 2.4 RT, amplification

A suspension of a single cell in phosphate-buffered saline (PBS) at a concentration of 2 × 105 cells/mL was introduced onto a microfluidic chip utilizing the Singleron Matrix^®^ single cell processing system. Following this, the barcode-labeled beads were retrieved from the chip, and the messenger RNA (mRNA) captured by the beads was reverse transcribed to generate complementary DNA (cDNA), which was subsequently amplified via polymerase chain reaction (PCR). The amplified cDNA was then fragmented and ligated to a sequencing adapter. The single-cell RNA sequencing (scRNA-seq) library was prepared in accordance with the GEXSCOPE^®^ single cell RNA library kit protocol (Singleron). The library was diluted to a concentration of 4 nM, pooled, and subjected to sequencing on an Illumina NovaSeq 6,000 platform with 150 bp paired-end reads. Subsequent data preprocessing steps included cell barcode extraction, genome read alignment, and unique molecular identifier (UMI) counting. Single-cell analysis was primarily conducted using Seurat 4.0, encompassing processes such as cell and gene selection, variance regression, data normalization, multi-sample integration, cell clustering, cluster-level marker gene identification, and data visualization. The annotation of epididymal cell types is determined by the identification of known epididymal cell marker genes obtained from prior literature.

### 2.5 Data analysis

Utilizing single cell sequencing data, we extracted pertinent gene information from the GeneBank and GeneCards databases to preliminarily deduce the potential biological function of a specific cell subgroup. Subsequently, we conducted a thorough literature search on PubMed to either substantiate our inference or propose novel perspectives.

## 3 Result

### 3.1 Changes in the grouping and proportion of cells in the epididymal head and tail in 5-week and 10-week mice

**FIGURE 1:**
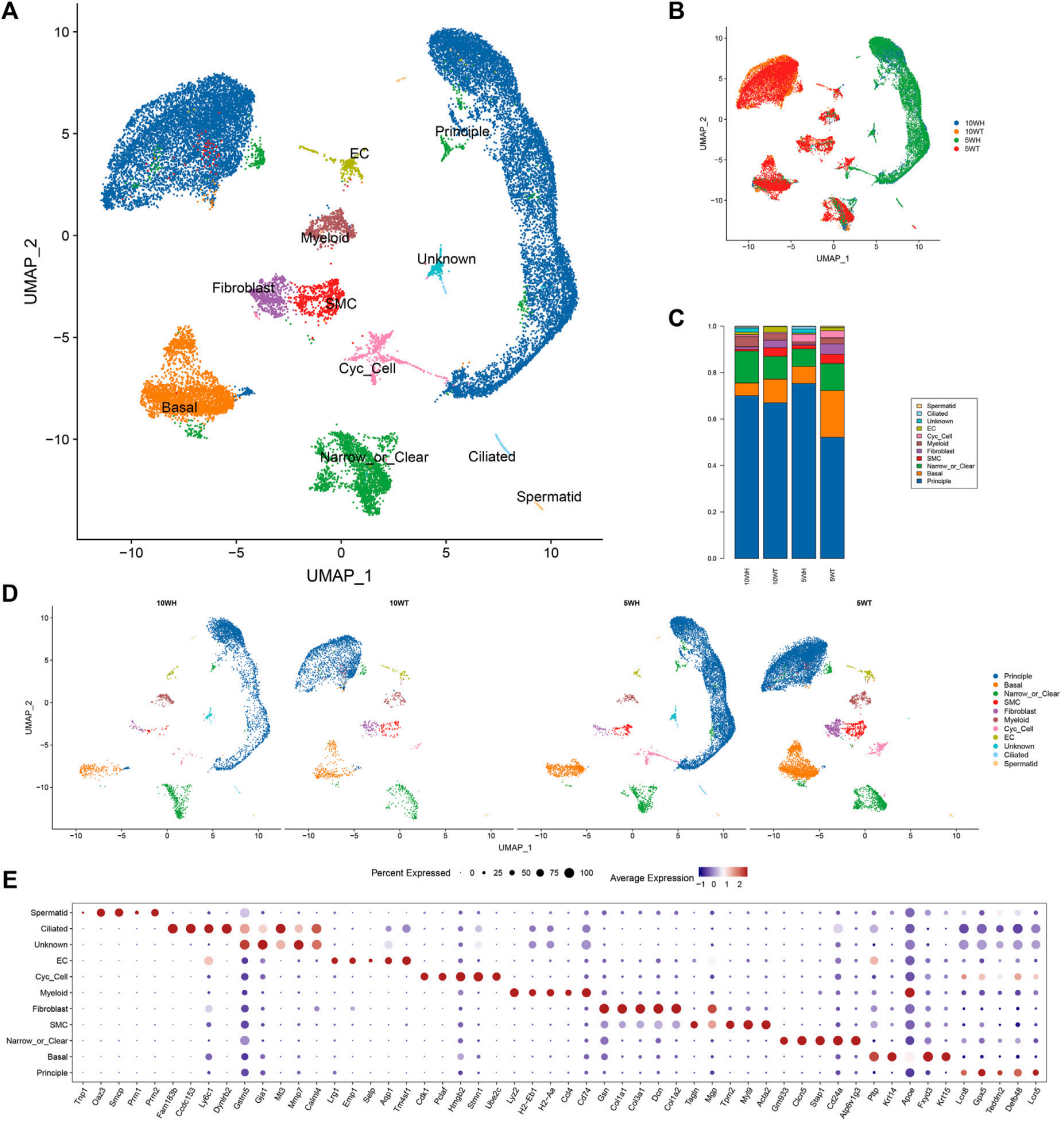
The results of single-cell sequencing of epididymis head and tail of 5 and 10 W mice. **(A)** UMAP diagram of epididymal single-cell clustering; **(B)** Single-cell clustering UMAP map classified by group; **(C)** The proportion of each cell type in different groups; **(D)** The UMAP maps of epididymal single cells in each group were clustered respectively; **(E)** TOP5 expression marker gene of each cell type.

Using the scRNA-seq methodology, quality control measures were implemented to remove reads contaminated with cell-free RNA, resulting in the acquisition of 27,896 single-cell data points from four distinct sample groups: 5-week mouse epididymal head (5 W H), tail (5 W T), 10-week mouse epididymal head (10 W H), and tail (10 W T) (Figure 1B). Cell clusters are classified into 10 distinct cell types, which include principle cell (PC), Basel cell (BC), Clear/Narrow cell (CC/NC), Smooth muscle cell (SMC), Fibroblast, Myeloid, Cycling cell (Cyc-cell), Endothelial cell (EC), Ciliated cell, a small number of spermatids, and an unidentified cell type (unknown) (Figure 1A). The main cells had the highest proportion, followed by clear cells and basal cells (Figure 1C). The top 10 differentially expressed genes were annotated within each cell cluster (Figure 1E). The highest proportion of principal cells in the caput and cauda epididymides was approximately 60%-80%, with clear cells and basal cells following suit (Figure 1C). Ciliated cells were exclusively present in the head of the epididymis, with a higher expression at 5 weeks compared to 10 weeks (Figure 1D). Additionally, the proportion of smooth muscle cells, fibroblasts, and endothelial cells in the tail exceeded that in the head.

### 3.2 The characteristics of principal cell subsets and their distinctions pre- and post-maturation

**FIGURE 2:**
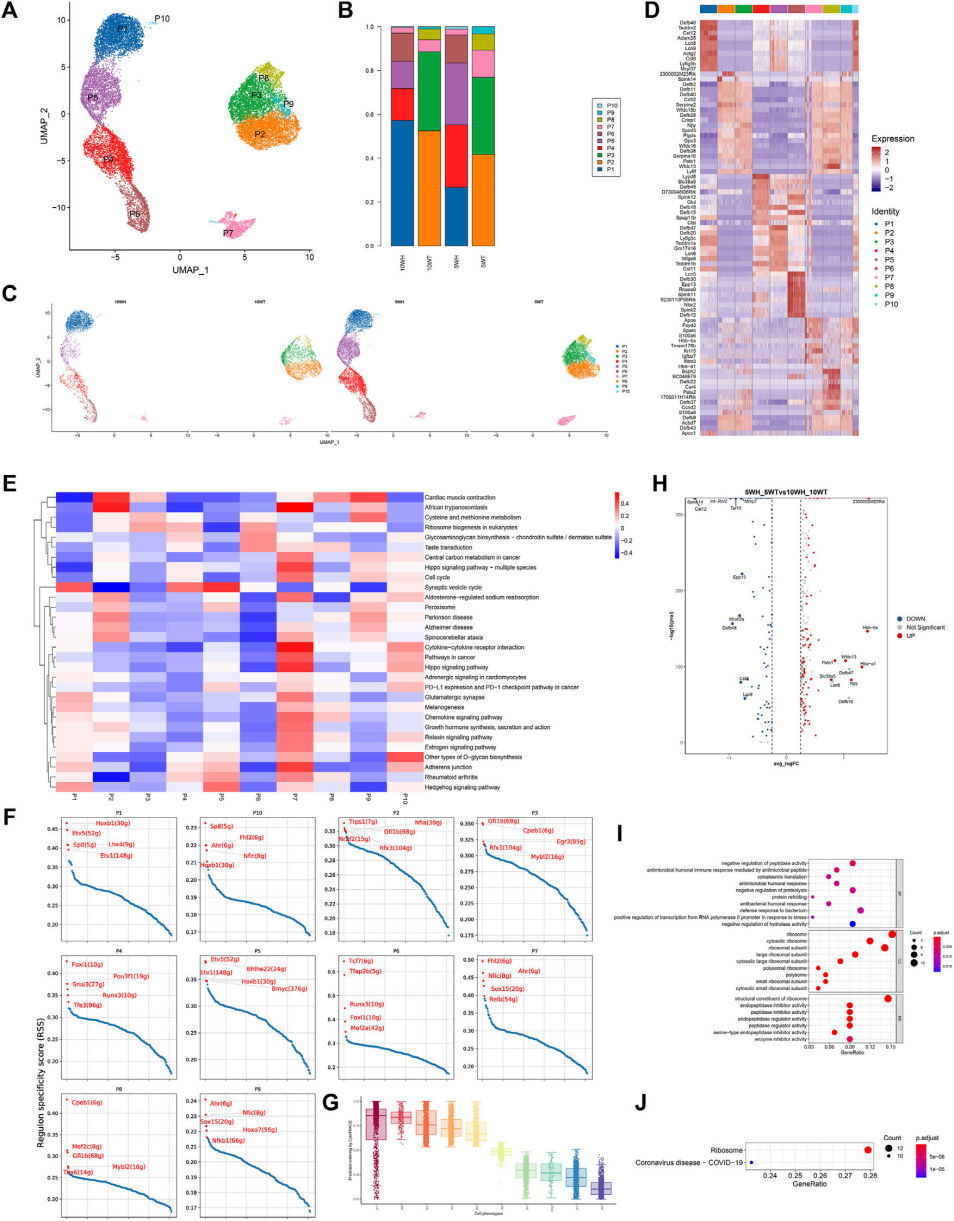
Characteristics of epididymal main cell subsets and differences before and after principal cell sexual maturity. **(A)** The secondary clustering of principal cells was divided into 10 subtypes: **(B)** The proportion of each subtype in the head and tail of the epididymis at 5 W and the head and tail of the epididymis at 10 W; **(C)** UMAP graph colored according to the sample; **(D)** Different cluster differential gene list; **(E)** GSEA enrichment analysis of each subtype of principal cell; **(F)** Each principal cell subtype transcription factor-specific scatter plot highlights the highest top5 regulon; **(G)** The box plot distribution of cytotrace scores of cell types; **(H)** The heat map of top upregulated genes and top downregulated genes sorted by average _ logFC between 5 W epididymis vs. 10 W epididymis; **(I)** GO enrichment analysis bubble diagram based on 5 weeks vs. 10 weeks upregulated differential genes; **(J)** KEGG enrichment analysis bubble diagram of upregulated differential genes in 5 weeks vs. 10 weeks.

The principal cells were categorized into 10 subgroups based on marker genes (Figure 2A), with P1, P4, P5, P6, and P10 exclusively present in the epididymis head, and the majority of P2, P3, P8, and P9 found only in the epididymis tail (Figures 2B, C). A heat map was utilized to display the marker genes of the 10 subgroups of main cells, such as sparc for P7 and Ctsl for P10 (Figure 2D). For instance, utilizing competitive gene set enrichment analysis (GSEA), it was observed that the principle cell P5 subtype exhibited significant activity in the Synaptic vesicle cycle and Hedgehog signaling pathway, while the p7 subtype demonstrated heightened activity in Adherens junction, Cytokine-cytokine receptor interaction, Chemokine signaling pathway, Cell cycle, Growth hormone synthesis, secretion and action, Relaxin signaling pathway, Estrogen signaling pathway, and Hippo signaling pathway (Figure 2E). The CytoTRACE analysis revealed that P7 exhibits the greatest differentiation potential, suggesting it may serve as the initial stage for master cell differentiation (Figure 2G). This analysis highlights the significant role of the P7 subgroup in the development of epididymal principal cells. Furthermore, the specific transcription factors of the main cell subgroups were determined utilizing pySCENIC (Figure 2F).

The predominant cell subtypes exhibited notable alterations in both pre-sexual and post-sexual maturation. Specifically, from 5 weeks to 10 weeks, there was a significant increase in the proportion of P1, P2, and P4 cells from the epididymal head (H), accompanied by a significant decrease in the proportion of P5 and P10 cells. Analogous changes were observed in the cauda epididymidis (T), where the proportion of P2 and P3 cells increased while the proportion of P7, P8, and P9 cells decreased (Figure 2C). The findings from the analysis of gene expression differences in epididymal principal cells at 5 and 10 weeks post-sexual maturity indicate a downregulation of genes such as 2300002M23Rik, Hbb − bs, Hba − a1, Npy, Wfdc13, and an upregulation of genes such as Spink14, Cst12 (Figure 2H). The results of gene ontology analysis indicated that the upregulated genes in epididymal principal cells following sexual maturation are implicated in various biological processes, including antimicrobial humoral immune response mediated by antimicrobial peptide, antimicrobial humoral response, defense response to bacterium, ribosome, and peptidase inhibitor (Figure 2I). Additionally, KEGG enrichment analysis revealed that these cells are associated with the Coronavirus disease–COVID-19 signaling pathway post-sexual maturation (Figure 2J).

**FIGURE 3:**
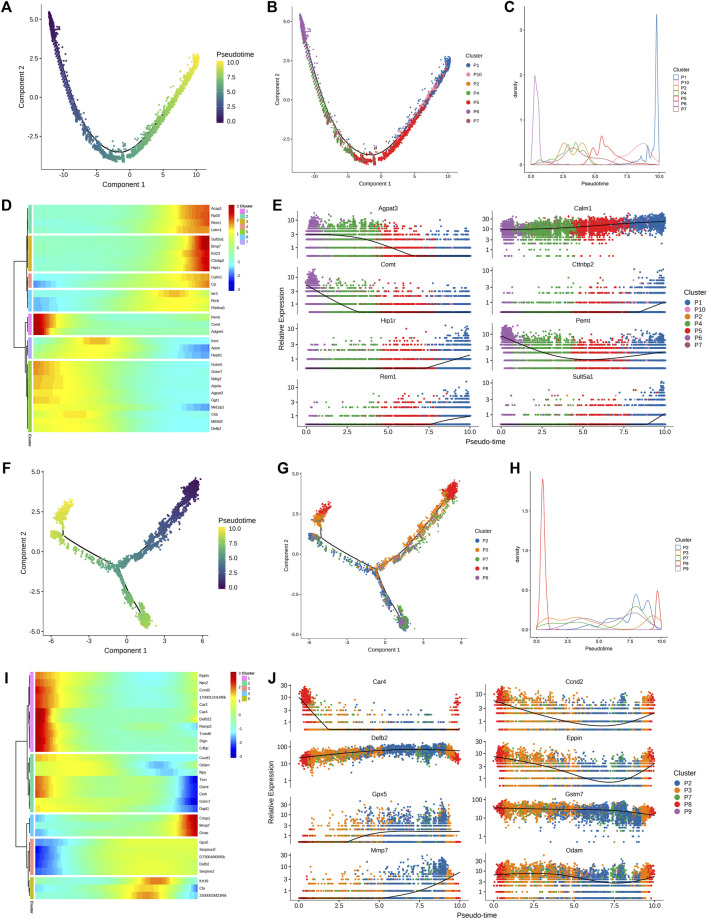
Quasi-timing analysis of the principal cells **(A)** All the main cells of the epididymal head were sorted by quasi-timing. The order of the time indicates the order of the pseudo time of differentiation; **(B)** The quasi-time analysis was colored according to the cell type. Different colors represent different cell types, reflecting the differentiation pseudo-time of different epididymal head main cell subtypes; **(C)** The abscissa is the pseudo-time, and the ordinate is the density of the number of cells at different time points. Different colors represent the density distribution of different epididymal head main cell types with pseudo-time changes; **(D)** Display the dynamic changes of gene expression with pseudo-time changes, the abscissa from left to right is the time from small to large, the ordinate is the gene, and each point represents the average expression of the specified gene at the specified pseudo-time; **(E)** shows genes that are differentially expressed over pseudo-time. The abscissa from left to right is the pseudo-time from small to large, the ordinate is the expression of the gene, and different colors indicate different cell types; **(F–J)** Quasi-timing analysis of cauda epididymides main cells sorts similar to **(A–E)**.

Following this, a quasi-sequential sorting analysis was conducted on all master cells from the epididymal head and tail. The findings indicated that the initiation point of the quasi-sequential trajectory for epididymal head master cells (WH-P) was at P6 (Figures 3A–C). Subsequently, various genes that exhibited significant changes along the quasi-temporal trajectory were identified and analyzed (Figures 3D, E). The study revealed that genes associated with protein synthesis, transport, and catalysis (e.g., Acap1, Rp3l3 expression) exhibited an increase along a quasi-temporal trajectory. Conversely, genes related to the cytoskeleton and cell morphology (e.g., Rem1, Cttnbp2 expression) showed an increase with pseudo-timing changes. Additionally, genes linked to mitochondrial morphology and cell viability (e.g., Letm1 expression) also increased with pseudo-timing changes. The upregulation of genes associated with epididymal function, including Bmp7, was observed to increase in accordance with changes in pseudo-temporal sequencing. Similarly, genes related to sperm motility and maturation, such as Adgre5 and Calm1, also exhibited increased expression with changes in pseudo-temporal progression. The initiation point of the pseudo-temporal trajectory of the main cells in the epididymal tail, denoted as WT-P, was identified as the P8 subgroup (see Figures 3F–H). Genes associated with the suppression of male reproductive function, such as Eppin and Npc2, exhibited a decrease in expression levels with quasi-sequential alterations. Conversely, genes involved in epididymal and sperm maturation, such as Crisp1, Gnas, Krt19, and Clu, as well as genes responsible for sperm protection, such as Gpx5, demonstrated an increase in expression levels with quasi-temporal changes.

### 3.3 The study examines the distinct characteristics of clear/narrow cells subsets and the variations in main cells before and after sexual maturity

**FIGURE 4:**
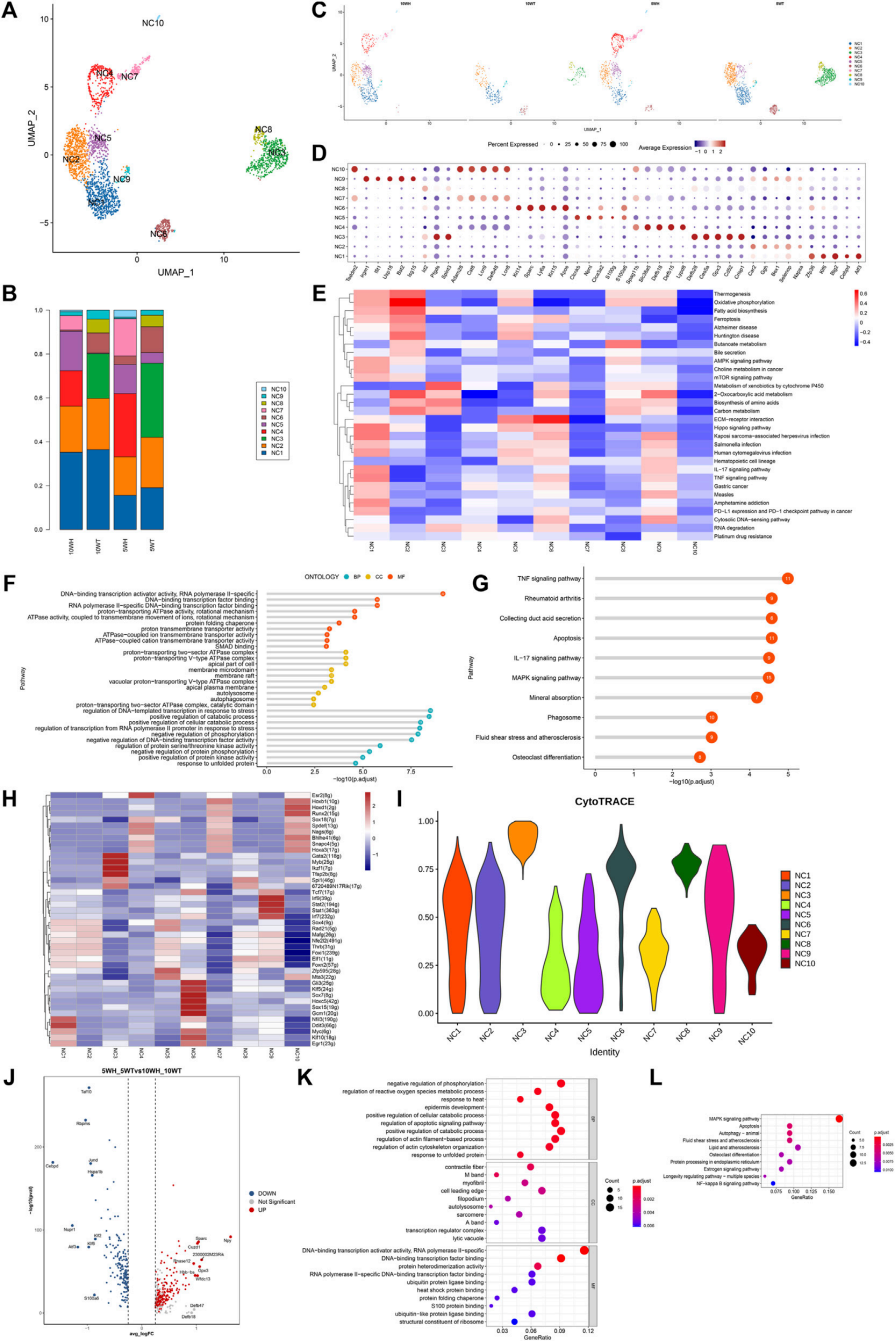
The characteristics of epididymal clear cell subsets and the differences before and after the sexual maturation of clear cells. **(A)** The secondary clustering of clear cells was divided into 10 subtypes; **(B)** The proportion of each subtype in the head and tail of the epididymis at 5 W and the head and tail of the epididymis at 10 W; **(C)** UMAP graph colored according to the sample; **(D)** After the differential gene list of different cell types or clusters was sorted in descending order according to average _ logFC, the first five genes were selected for bubble mapping, and duplicate genes were removed in the mapping. **(E)** GSEA enrichment analysis of clear cell subtypes; **(F)** GO enrichment analysis of NC1 high expression genes; **(G)** NC1 high expression gene KEGG analysis; **(H)** Calculate the AUC matrix of cell type by mean value, and the AUC matrix clustering heat map of top5 regulon in each cell type; **(I)** The violin plot distribution of cytotrace scores for each cell type; **(J)** Volcano plots of top upregulated genes and top downregulated genes sorted by average _ logFC between 5 W epididymis vs. 10 W epididymis groups; **(K)** Based on the GO enrichment analysis of upregulated differential genes at 5 weeks vs. 10 weeks, the common or unique gene display of each pathway was analyzed; **(L)** KEGG enrichment analysis of upregulated differential genes at 5 weeks vs. 10 weeks showed common or unique genes in each pathway.

The clear/narrow cell machinery was subdivided into 10 subgroups, as depicted in Figure 4A. Subgroups NC1, NC2, NC5, NC6, and NC9 exhibited expression in both the caput and cauda of the epididymis, while NC4, NC7, and NC10 were exclusively expressed in the caput, and NC3 and NC8 were solely expressed in the cauda (Figures 4B, C). Gene expression patterns for each subtype of clear cells are illustrated in Figure 4D. Analysis using GSVA revealed that NC1 displayed heightened activity in the AMPK pathway, mTOR signaling pathway, Hippo signaling pathway, and TNF signaling pathway. NC2 primarily emphasized thermogenesis, oxidative phosphorylation, fatty acid biosynthesis, ferroptosis, two-oxocarboxylic acid metabolism, and carbon metabolism. On the other hand, NC3 indicates a correlation between the metabolism of xenobiotics by cytochrome P450 and amino acid biosynthesis. Additionally, NC6 showed a significantly higher impact on ECM-receptor interaction, while NC8 exhibited increased activity in the butyric acid metabolic pathway (Figure 4E).

To further investigate the disparity in gene expression of epididymal clear cells in mice before and after sexual maturity, an analysis was conducted on the gene expression differences and GO, KEGG enrichment of 5-week and 10-week epididymal head and tail clear cells (Figures 4J–L). The GO analysis revealed that genes highly expressed in epididymal clear cells post-sexual maturity were associated with response to heat, regulation of reactive oxygen species metabolic process, regulation of apoptotic signaling pathway, autolysosome, heat shock protein binding, and other biological processes. The results of KEGG enrichment analysis indicated that epididymal clear cells following sexual maturation are associated with activation of the MAPK signaling pathway, apoptosis, autophagy, estrogen signaling pathway, and NF-kappa B signaling pathway.

The composition of clear cell subsets exhibited a notable shift following sexual maturation, particularly with an increase in the proportion of the NC1 subtype in the head and tail of the epididymis. Given the elevated presence of NC1 in the 5-week and 10-week epididymis segments, as well as the heightened activity of associated pathways in the GSVA analysis, further investigation through GO and KEGG enrichment analysis was conducted to elucidate the functional role of NC1. The results of gene ontology (GO) analysis indicated that genes with high expression levels of NC1 are associated with DNA binding transcriptional activator activity, ATPase activity, DNA transcriptional regulation in response to stress, and other biological processes (Figure 4F). Additionally, Kyoto Encyclopedia of Genes and Genomes (KEGG) analysis revealed that NC1 high expression genes are involved in pathways such as TNF signaling, apoptosis, IL-17 signaling, MAPK signaling, and phagosome pathways (Figure 4G). Subgroup-specific transcription factors for clear cells were determined using pySCENIC (Figure 4H). NC3 exhibits the greatest potential for differentiation, as indicated in Figure 4I.

**FIGURE 5:**
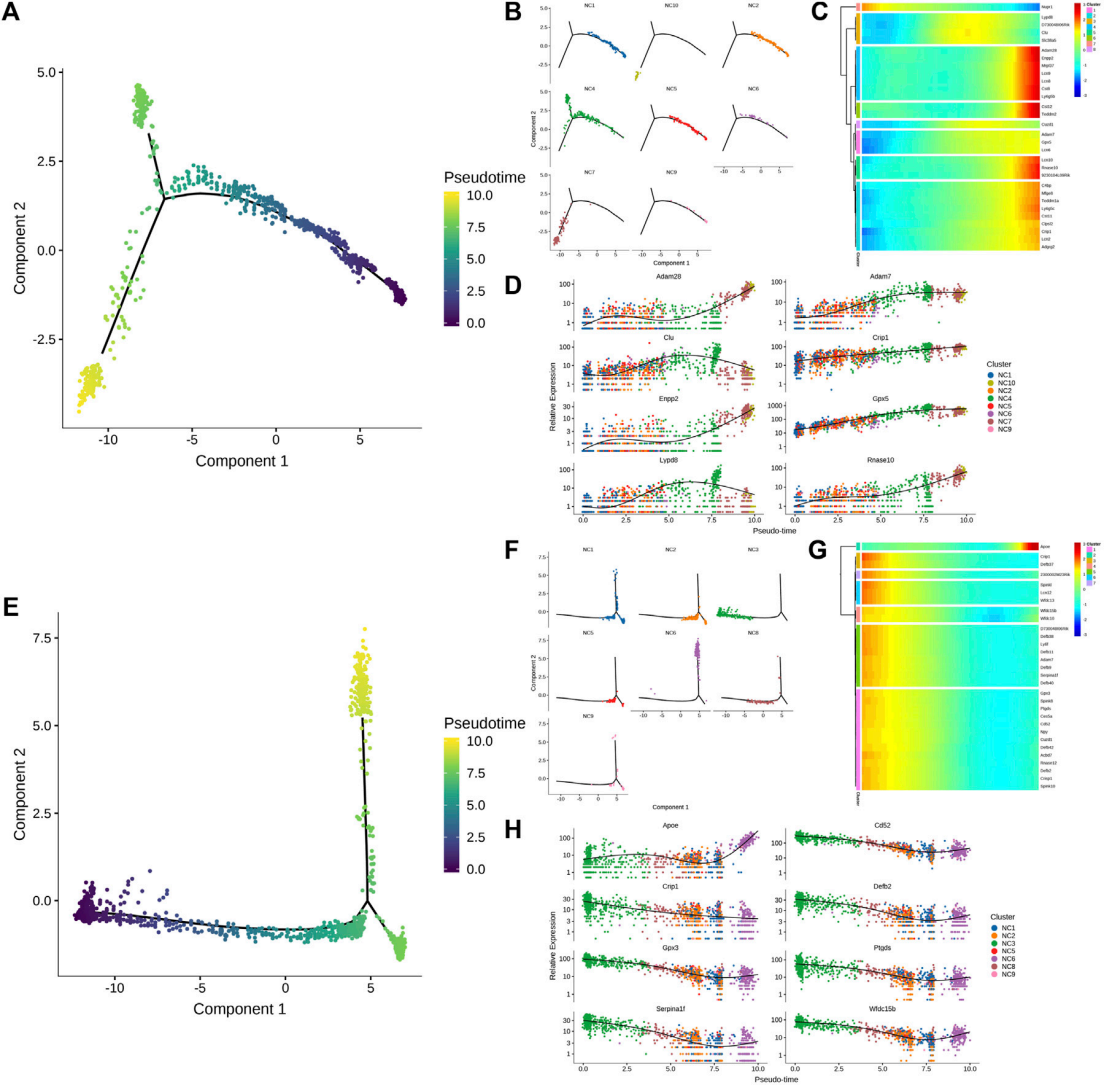
Quasi-timing analysis of the main cells. **(A)** Quasi-sequential sorting of all epididymal head clear cells. The order of the time indicates the order of the pseudo time of differentiation; **(B)** The distribution map of each cell type in the pseudo-timing trajectory; **(C)** The density distribution of different types of epididymal cauda clear cells with pseudo-time changes; **(D)** Display the dynamic changes of gene expression with pseudo-time changes; **(E–H)** Epididymal cauda clear cell pseudo-time series analysis, sort similar to **(A–D)**.

The clear cell of the epididymal head, following a pseudo-sequential trajectory denoted as WH-NC, originates from NC1 as shown in Figures 5A, B. Through screening various genes that exhibit significant changes along the quasi-temporal trajectory, we conducted an analysis, as illustrated in Figures 5C, D. Notably, the expression of Nupr1 decreased in correlation with the developmental trajectory alteration. The upregulation and subsequent downregulation of Lypd8 and other defense response genes in response to Gram-negative bacteria, as well as the similar pattern observed for Clu, a gene associated with apoptosis, were noted. Additionally, the expression of Slc38a5, implicated in the creation of an acidic environment within the epididymal lumen, exhibited an increase. Furthermore, genes involved in sperm maturation, including Adam28, Adam7, and Rnase10, were found to be upregulated, along with genes such as Gpx5, which play a role in protecting sperm. The initiation of the pseudo-sequential trajectory of the epididymal cauda clear cell (WT-NC) commences with the NC3 subgroup as shown in Figures 5E, F. The expression of Apoe diminishes as pseudo-timing progresses. Similarly, the levels of Cd52, Defb2, Gpx3, Ptgds, and other genes decrease in accordance with the pseudo-time sequence (Figures 5G, H).

### 3.4 The study examines the characteristics of basal cell subsets and explores the disparities in these subsets before and after sexual maturation

**FIGURE 6:**
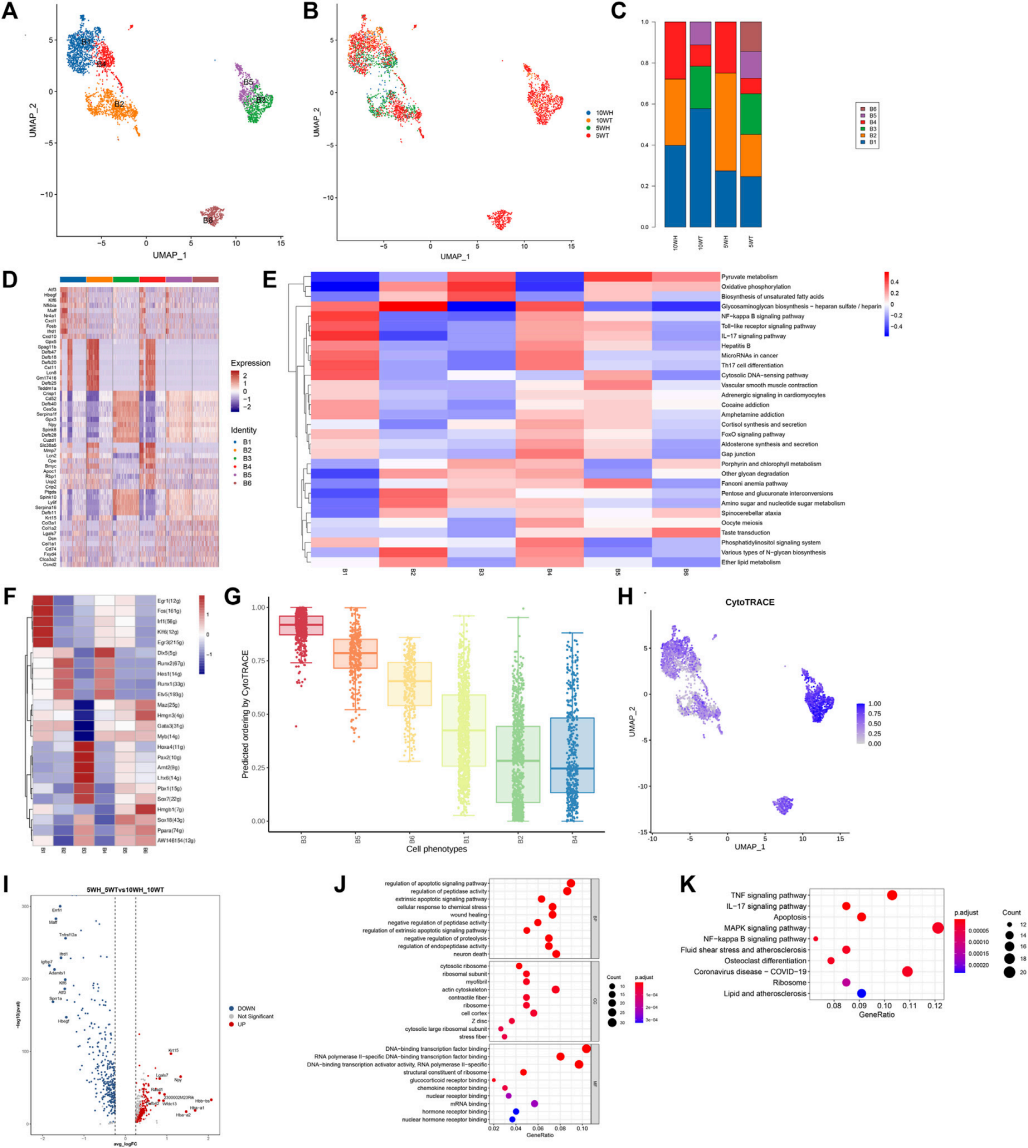
Characteristics of epididymal basal cell subsets and differences before and after sexual maturity of basal cells. **(A)** The secondary clustering of basal cells was divided into 6 subtypes; **(B)** According to the sample coloring of the UMAP diagram; **(C)** The proportion of each subtype in the head and tail of the epididymis at 5 W and the head and tail of the epididymis at 10 W; **(D)** The differential gene list of different cell types or clusters was selected for heat map drawing; **(E)** GSEA enrichment analysis of each subtype of basal cells; **(F)** top5 regulon AUC matrix clustering heat map in each cell type; **(G)** The box plot distribution of cytotrace scores of cell types; **(H)** The UMAP plot was colored by Cytotrace score; **(I)** The volcano map of top up-regulated genes and top down-regulated genes sorted by avg _ logFC between 5 W epididymis vs 10 W epididymis; **(J)** GO enrichment analysis heat map based on 5 weeks vs 10 weeks up-regulated differential genes; **(K)** KEGG enrichment analysis heat map of up-regulated differential genes at 5 weeks vs 10 weeks.

Basal cells were categorized into six subgroups based on marker genes, as depicted in Figure 6A. Among these subgroups, B3, B5, and B6 were exclusively localized in the epididymal tail, as shown in Figures 6B, C. Over the period from 5 weeks to 10 weeks, there was a notable increase in the proportion of B1 and B4 cells in the epididymal head (H), accompanied by a significant decrease in the proportion of B2 cells. Conversely, in the epididymal tail (T), there was an increase in the proportion of B1 and B4 cells and a decrease in the proportion of B2 and B6 cells, as illustrated in Figure 6C. Figure 6D displays the gene expression profiles of distinct subsets of principal cells. According to the results of competitive gene set enrichment analysis (GSEA), the basal cell B1 subtype exhibited elevated activity in the NF-κB signaling pathway, IL-17 signaling pathway, Th17 cell differentiation, and Toll-like receptor signaling pathway. Additionally, both the B1 and B4 subtypes demonstrated heightened activity in the Foxo signaling pathway and Hedgehog signaling pathway. Conversely, the B2, B3, and B6 subtypes displayed inhibition of the NF-κB signaling pathway, IL-17 signaling pathway, Th17 cell differentiation, Toll-like receptor signaling pathway, and Foxo signaling pathway. Moreover, the glycosaminoglycan biosynthesis pathways, particularly the B2 subtype heparin, exhibit elevated activity as depicted in Figure 6E. Additionally, CytoTRACE analysis indicates that the B3 subgroup demonstrates the highest potential for differentiation and may serve as the initiation point for basal cell differentiation, as illustrated in Figures 6G, H. Furthermore, the analysis reveals that the B1 and B4 subgroups are linked to inflammatory responses, whereas the B2, B3, and B6 subgroups are associated with anti-inflammatory responses. The specific transcription factors for each basal cell subgroup were determined using pySCENIC, as shown in Figure 6F.

To further investigate the differences in epididymal basal cells in mice before and after sexual maturity, a gene expression difference analysis and GO, KEGG enrichment analysis were conducted on basal cells from mice at 5 weeks and 10 weeks of age (Figures 6I–K). Following sexual maturity, the basal cells of mice exhibited upregulation of Maff, Errfi1, Tnfrsf12 a, Ifrd1, Igfbp7, Adamts1, Atf3, and Hbegf, while down-regulating krt15, Npy, Lgals7, and Defb42. GO analysis revealed that the highly expressed genes in epididymal basal cells after sexual maturity were associated with the regulation of apoptotic signaling pathways.

## 4 Discussion

**FIGURE 7:**
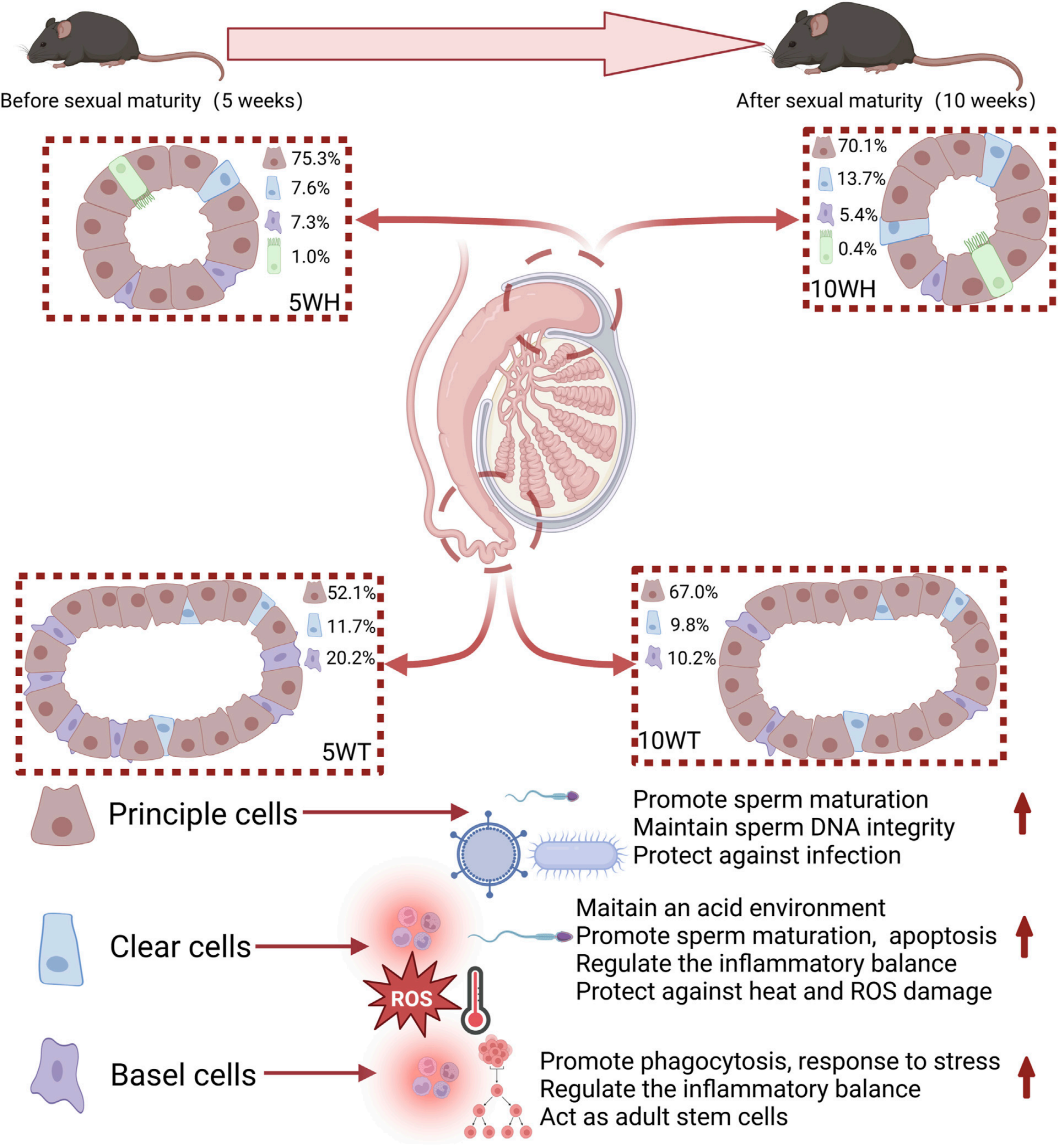
A graphical representation is provided in the figure illustrating the proportions of cell types and their respective functions throughout the developmental stages of mice from 5 weeks to 10 weeks. The red arrow signifies promotion.

This study represents the first instance of conducting single-cell sequencing on the mouse epididymis in both pre-sexual and post-sexual maturity. Specifically, single-cell sequencing was performed on the epididymal head and tail of mice at 5 and 10 weeks of age, resulting in the identification and clustering of 10 distinct cell clusters. Subsequent subgroup and pseudo-timing analyses were conducted on key cell types, including master cells, basal cells, and narrow/clear cells. A comparative analysis before and after sexual maturation revealed significant implications for sperm maturation, protection, antiviral immunity, and inflammatory response regulation. Figure 7 provides a visual representation of the key findings of the study, serving as a graphical summary of the article.

### 4.1 Type of cell in the epididymis

The duration of sperm production in mammals is contingent upon the length of the spermatogenic epithelial cell cycle, though the precise commencement and conclusion of spermatogenesis are challenging to ascertain (Shi et al., 2023). Research has shown that spermatozoa of 5-week-old C57BL/6J mice initially reached the tail region of the epididymis, yet were incapable of fertilizing oocytes (Mochida et al., 2019). It was not until day 37 that spermatozoa in the epididymal tail exhibited the capacity to fertilize oocytes. Adult male mice aged 10 weeks and older exhibit full sexual maturity, characterized by high fertilization potential of epididymal tail sperm (Mochida et al., 2019). Consequently, we chose the time points of 5 weeks and 10 weeks to represent stages before and after sperm maturation. Utilizing pseudo-timing analysis, we observed distinct gene expression patterns in the epididymal head, tail, and specific cell subtypes before and after sexual maturity, underscoring the critical role of the epididymis.

Epididymal epithelial cells are typically categorized into main cells, basal cells, and clear cells (Sylvie Breton et al., 2016). Leir et al. (2020) employed single-cell analysis to distinguish the epididymal head cell population from seven human epididymides aged 24-57 years, identifying eight distinct cell types: principal cells, basal cells, efferent duct cells, interstitial cells, clear cells, immune cells, sperm cells, and muscle cells. Shi et al. (2021) conducted a study that revealed distinct temporal and spatial distribution patterns of mitochondria and key genes in the head and tail of the epididymis in mice, suggesting potential functional differences between these regions. Consequently, the study employed separate sequencing of the head and tail of the epididymis. The research identified 10 distinct cell types within epididymal cells, including principal cells, basal cells, clear/narrow cells, smooth muscle cells, fibroblasts, myeloid cells, proliferative cells, endothelial cells, ciliated cells, and sperm cells. The presence of ciliated cells was limited to the head of the epididymis, with a higher proportion observed at 5 W (0.98%) compared to 10 W (0.43%). Girardet et al. (2020) demonstrated that these ciliated cells play a role in regulating antibacterial immunity and responding to fluctuations in the extracellular environment by activating the Hedgehog (Hh) signaling pathway.

### 4.2 Principal cells are integral in the processes of sperm maturation, antiviral defense, and anti-tumor immune responses following sexual maturation

The predominant cell type in the epididymal epithelium, known as the main cell, constitutes a significant proportion of the cellular composition in the epididymal head and tail of mice at different developmental stages. Leir et al. (2020) demonstrated that principal cells in the human epididymis express a variety of genes encoding proteins involved in various physiological functions, including serine protease inhibitors, antimicrobial peptides, cysteine-rich peptides, lipid transport proteins, and G protein-coupled receptors. The master cell is believed to play a crucial role in various functions related to sperm maturation (Leir et al., 2020), including regulation of epididymal lumen pH (Breton et al., 2019), prevention of infections and autoimmune reactions (Hall et al., 2007), protection against oxidative damage (Aitken, 2020), and control of semen coagulation and liquefaction (Clauss et al., 2011).

This study utilized mouse epididymis samples from both pre-and post-maturation stages (5 W and 10 W, respectively). The findings indicated a significant upregulation of gene expression, particularly Cst12, Cst8, and Lcn8, in the principal cells following sexual maturation. These genes are primarily associated with processes such as sperm maturation, lipid transport, and acrosome reaction (Chau and Cornwall, 2011; Li et al., 2005; Wen et al., 2021). Additionally, the study revealed an increased expression of genes related to peptidase inhibitors in the principal cells post-sexual maturation, which are known to play a crucial role in antiviral immunity (Pislar et al., 2020). Furthermore, analysis using the Kyoto Encyclopedia of Genes and Genomes (KEGG) revealed that epididymal principal cells are implicated in the Coronavirus disease (COVID-19) signaling pathway post-sexual maturation. These findings suggest a significant involvement of epididymal principal cells in sperm maturation following sexual maturity, with a notable emphasis on antiviral immune function. It is posited that the antiviral response observed may be linked to the regulation of peptidase activity by these principal cells. Pseudo-timing analysis revealed an upregulation of genes associated with epididymal and sperm maturation (e.g., Crisp1, Gnas, Krt19, Clu) as well as genes involved in sperm protection (e.g., Gpx5 expression) in conjunction with pseudo-timing alterations. Taylor et al.showed that GPX5 has a potent antioxidant effect that helps regulate reactive oxygen species levels in the epididymal microenvironment, protecting sperm from lipid peroxidation and maintaining DNA integrity (Taylor et al., 2013). These findings suggest that the principal cell plays a crucial role in both sperm maturation and protection.

Furthermore, the role of principal cells may be linked to a decreased risk of epididymal cancer, which represents only 0.03% of all male cancers, making it significantly less common than testicular tumors (Yeung et al., 2012). Currently, various mechanisms have been proposed for the prevention of epididymal cancer, including the presence of antioxidants and inactive oncogene products in the epididymis to prevent DNA damage and tumorigenesis (Yeung et al., 2012), the maintenance of tight junctions and the expression of growth inhibitory factors such as Rb1, Pten, DUSP6 to inhibit tumor proliferation (Voisin et al., 2019; Yeung et al., 2012), and the expression of anti-angiogenic factors like fibrin-5 and MMP to inhibit angiogenesis in non-angiogenic sites (Desai et al., 1998; Yeung et al., 2012), activation of antigen-presenting cells, γδT cells, helper CD4 + and cytotoxic CD8 + T cells by tumor cells to release IFN-γ, perforin and granzyme and inhibit angiogenesis and tumor proliferation (Voisin et al., 2018; Voisin et al., 2019), and the suppression of abnormal proliferation of epididymal cells through the action of Wnt, TGFα, and other factors to prevent their progression into tumors (Yeung et al., 2012). The results of this study indicate differential expression of sparc, Ctsl, and other genes in the primary P7/10 cell line. Sparc, a myocyte cytokine, has been shown to act as a tumor suppressor in multiple cancer types (Huang et al., 2022). Ctsl, highly expressed in lung cancer, breast cancer, and glioma, is implicated in the synthesis of elastin, collagen, and α-1 protease inhibitors, all of which are crucial for tumor progression. Targeted inhibition of Ctsl expression may hold promise for cancer therapy (Zhang et al., 2022). In the GSVA enrichment analysis, it was observed that P7/10 exhibited a strong association with cancer.

In summary, the epididymal principal cells of sexually mature mice exhibit a heightened capacity for promoting sperm maturation, antiviral immunity, and anti-tumor immunity compared to their pre-pubertal counterparts. Specifically, the enhanced sperm function facilitated by these principal cells post-sexual maturity is associated with upregulated gene expression, including Cst12, Cst8, and Lcn8. The increased antiviral immune function is linked to improved peptidase regulation by host cells, while the anti-tumor immunity is primarily influenced by the P7/P10 subtype of principal cells.

### 4.3 Clear cells potentially have a significant function in safeguarding sperm and modulating the pH levels within the epididymis

Narrow cells are limited to the initial segment of the human epididymis and are absent in other regions (Sullivan et al., 2019). Clear cells, on the other hand, are present in the head, body, and cauda of the epididymis, with a notably higher concentration in the cauda compared to the tectum (Shum et al., 2011). Despite this difference, both cell types express vacuolar proton pump V-ATPase, essential for acidifying the lumen and preserving sperm viability prior to ejaculation (Sullivan et al., 2019). Rinaldi categorizes clear cells into three distinct subcellular populations, suggesting their involvement in modulating energy metabolism and membrane transport (Rinaldi et al., 2020). Prior research has demonstrated that the primary role of clear cells is to create an acidic lumen environment to maintain sperm quiescence prior to mating (Leir et al., 2020), transport proteins to sperm via epididymal extracellular vesicles or nanotubes (Battistone et al., 2020), and regulate the immune response to safeguard the epididymis (Battistone et al., 2020).

The study’s analysis revealed that clear cells play a crucial role in safeguarding sperm or the epididymis from elevated temperatures and reactive oxygen species, as well as in maintaining the pH balance within the epididymis. Gene Ontology analysis indicated that genes highly expressed in epididymal clear cells post-sexual maturation are involved in responding to heat stress and regulating the metabolic process of reactive oxygen species. Prior research has demonstrated that reactive oxygen species (ROS) are integral to the process of sperm maturation and capacitation, necessitating a delicate equilibrium between ROS generation and scavenging in the vicinity of sperm cells (Drevet, 2006; Chabory et al., 2010). The enzyme glutathione peroxidase (GPX) is known to play a significant role in this process (Drevet, 2006). The present study observed a positive correlation between the expression of GPX5 in epididymal clear cells and the progression of the pseudo-time sequence. Furthermore, it was found that epididymal epithelial clear cells are involved in safeguarding sperm and the epididymis from thermal injury. Li ZJ et al. demonstrated an increase in the number of clear cells in the epididymis of boars subjected to heat treatment (Li et al., 2017). Furthermore, the Gene Set Enrichment Analysis (GSEA) conducted in this study revealed that NC1 exhibited heightened AMPK signaling pathway activity, while NC2 showed increased oxidative phosphorylation activity. These findings suggest that clear cells in the epididymal epithelium play a crucial role in regulating the pH balance within the epididymal cavity by facilitating ATP production and hydrogen ion transport through clear cell H + -ATPase.

Furthermore, following sexual maturity, there is an observed enhancement in the immune function of epididymal clear cells compared to pre-sexual maturity. This enhancement is attributed to the activation of the NF-kappa B signaling pathway in clear cells. The NC1 subtype is identified as a key factor contributing to the heightened immune function of epididymal clear cells, with the NC1 subtype mTOR signaling pathway, Hippo signaling pathway, and TNF signaling pathway displaying increased activity. Additionally, sexually mature clear cells exhibit enrichment in TNF signaling, apoptosis, IL-17 signaling, and MAPK signaling pathways when compared to their pre-sexual maturity counterparts. Among them, mTOR is involved in antibacterial immunity (Abdel-Nour et al., 2014). Clear cells have the capacity to establish a balance between pro-inflammatory and anti-inflammatory responses through modulation of TNF signaling, apoptosis, IL-17 signaling, MAPK signaling, and Hippo signaling pathways (Herjan et al., 2018; Huang et al., 2010; Messer, 2017; Mia and Singh, 2022; Zelova and Hosek, 2013). This phenomenon implies that following sexual maturation, the altered function of NC1 results in clear cells assuming a more prominent role in antibacterial activities and inflammation regulation. The observed rise in the proportion of NC1 post-sexual maturation further supports this assertion.

### 4.4 Basal cells play a role in the modulation of inflammatory and stress responses

Basal cells in mammals are individual monolayer cells located at the base of the epididymal epithelium, serving as a distinctive marker of the epididymis. These entities are recognized for their roles in various physiological functions, including clearance (Suzuki and Racey, 1976), passage through the blood-epididymis barrier as an intracavitary sensor (Sylvie Breton et al., 2016), involvement in cell adhesion, cytoskeleton arrangement, ion transport, cell signal transduction, regulation of intracavitary pH and inflammatory response (Breton et al., 2019), maintenance of the structural integrity of the blood-epididymis barrier (Cyr et al., 2007), adult stem cell functions (Mandon et al., 2015), and participation in male resistance to reactive oxygen species and prostaglandin secretion (Mandon et al., 2015). Leir SH and colleagues analyzed the single-cell atlas of the epididymis, revealing an association between basal cells and tumor-associated calcium transduction (Leir et al., 2020).

This study posits a potential relationship between basal cells and inflammatory response. Specifically, the analysis of basal cell subsets revealed that B1 and B4 subsets were linked to inflammatory response, whereas B2, B3, and B6 subsets were associated with anti-inflammatory response. The highly expressed genes in epididymal basal cells following sexual maturity impact biological structures and processes, including stress fiber and glucocorticoid receptor binding. Inflammation is triggered by alarm molecules released in response to tissue stress or injury. Belardin, LB et al. demonstrated that vas deferens ligation resulted in an epididymal stress response, characterized by strong labeling of epididymal epithelial basal cells with the pro-inflammatory factor P2Y14 and subsequent immune cell recruitment (Belardin et al., 2022). Additionally, the study found differential expression of various transcriptional activators (Atf3, Klf6, Maff, Nr4a1) and immune-related factors (Hbegf, Nfkbia, Cxcl1, Cxcl10) by B1 cells. Several transcription factors, including Atf3, Klf6, Maff, and Nr4a, have been identified as key players in regulating various cellular processes and inflammation-related pathological conditions. Studies have demonstrated the significance of dysregulation of these factors in the development of inflammatory diseases and the modulation of immune cell balance during inflammation (Blank, 2008; Syafruddin et al., 2020; Crean and Murphy, 2021; Chen et al., 2022). Hbegf facilitates smooth muscle cell proliferation via EGFR, ERBB2, and ERBB4, and is involved in macrophage-mediated cell proliferation (Jessmon et al., 2009). The Nfkbia-encoded protein interacts with the REL dimer to suppress the NF-kappa-B/REL complex, which plays a role in the inflammatory response (Li and Verma, 2002). Elevated B1 scores in GSVA’s 'NF-κB signaling pathway', 'IL-17 signaling pathway', 'Toll-like receptor signaling pathway', and other pathways suggest a potential role of basal cells in epididymal immunity, particularly in the inflammatory response.

Following sexual maturation, there is a gradual enhancement in the regulatory inflammatory function of epididymal basal cells compared to their pre-sexual maturity state. Post-sexual maturity, basal cells exhibit enrichment in TNF signaling pathway, IL-17 signaling pathway, apoptosis, and NF-kappa B. These basal cells, upon sexual maturation, demonstrate varied pro-inflammatory and anti-inflammatory effects by activating these signaling pathways to maintain a balance between pro-inflammatory and anti-inflammatory responses. Prior research has demonstrated that epididymal basal cells and mononuclear macrophages exhibit similarities in antigen expression and function, thereby contributing significantly to the immune microenvironment of the epididymis (Seiler et al., 1999; Yeung et al., 1994).

Furthermore, the role of mouse epididymal basal cells in modulating stress response exhibited a gradual increase following sexual maturation. Npy demonstrated significant expression levels in 10-week-old mice, as evidenced by Reichmann and Holzer (2016) research highlighting the pivotal role of Npy in stress response. These findings suggest that epididymal basal cells may modulate stress response via Npy following sexual maturation. Furthermore, GO enrichment analysis revealed that the differentially expressed genes in 10-week-old mice are associated with biological processes related to stress fibers and glucocorticoid receptors. Silva et al. demonstrated that glucocorticoids and stress fibers are potentially essential factors in the epididymis for facilitating sperm maturation (Silva et al., 2010).

## 5 Summary

The study found that cells in the epididymis have distinct roles in sperm maturation, inflammation regulation, and antiviral defense. Each type of cell within the epididymis exhibits distinct functions, with principal cells playing a role in maturation, antiviral and anti-tumor immunity, as well as regulation of pH levels. Transparent/narrow cells are involved in sperm protection and inflammatory response regulation, while basal cells primarily regulate inflammatory and stress responses. The study results indicate significant heterogeneity among epididymal cell types, with each subgroup demonstrating unique functionalities. The distribution of the head and tail exhibited variation, as evidenced by the pseudo-temporal analysis which highlighted distinctions in their development and differentiation. For instance, the exclusive presence of the principal cell P1 subgroup in the epididymal head suggests its role in maintaining sperm motility and facilitating sperm maturation. Additionally, the clear cell NC4, found solely in the caput epididymis, demonstrates high expression levels in the regulation of pH and protection of sperm-related genes.

This study examined cytological changes in epididymal cells before and after maturation at 5 and 10 weeks. There were spatial differences in the proportion and function of cells, with ciliated cells only found in the epididymal head and more prevalent at 5 weeks compared to 10 weeks. The study showed that sperm maturation improves before and after epididymal maturation, enhancing antiviral and bacterial infection resistance, and gradually improving regulation of epididymal lumen pH, inflammation, and stress response. This study offers new insights into the immune function of epididymal epithelial cells and their role in sperm maturation and immunity, potentially leading to new treatments for male infertility.

## Data Availability

The datasets presented in this study can be found in online repositories. The names of the repository/repositories and accession number(s) can be found below: https://www.cncb.ac.cn/, CRA015988.
